# 2,2′,5,5′-Tetra­chloro-*N*,*N*′-diethyl-*N*,*N*′-[benzene-1,3-diylbis(methyl­ene)]dibenzene­sulfonamide

**DOI:** 10.1107/S1600536811045326

**Published:** 2011-12-03

**Authors:** Ejaz, Islam Ullah Khan, Tahir Ali Sheikh, William T. A. Harrison

**Affiliations:** a Materials Chemistry Laboratry, Department of Chemistry, GC University, Lahore 54000, Pakistan; b Department of Chemistry, University of Aberdeen, Meston Walk, Aberdeen AB24 3UE, Scotland

## Abstract

In the title compound, C_24_H_24_Cl_4_N_2_O_4_S_2_, the dihedral angles between the central benzene ring and the pendant rings are 58.09 (10) and 62.59 (10)°. The dihedral angle between the pendant rings is 81.64 (9)°. Both sulfonamide groups lie to the same side of the central ring but the C—S—N—C torsion angles [73.09 (16) and −117.35 (14)] and S—N—C—C torsion angles [−143.80 (14) and −111.45 (16)°] differ significantly for the two pendant chains. The N atoms are close to planar (bond angle sums = 356.4 and 359.5°). In the crystal, weak C—H⋯O and C—H⋯Cl inter­actions link the mol­ecules.

## Related literature

For related structures, see: Ejaz *et al.* (2011*a* ▶,*b* ▶).
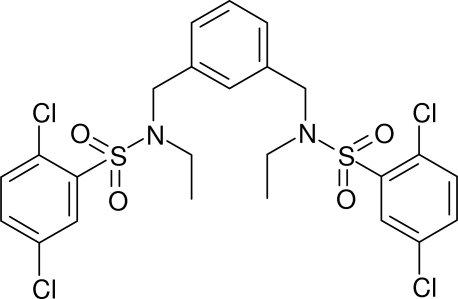


## Experimental

### 

#### Crystal data

C_24_H_24_Cl_4_N_2_O_4_S_2_
*M* *_r_* = 610.37
Triclinic, 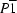

*a* = 8.0396 (2) Å
*b* = 11.1512 (3) Å
*c* = 15.5723 (3) Å
α = 87.454 (1)°
β = 83.378 (1)°
γ = 87.995 (1)°
*V* = 1384.77 (6) Å^3^
*Z* = 2
Mo *K*α radiation
μ = 0.61 mm^−1^
*T* = 296 K
0.50 × 0.35 × 0.30 mm

#### Data collection

Bruker APEXII CCD diffractometer
25271 measured reflections
6905 independent reflections
5380 reflections with *I* > 2σ(*I*)
*R*_int_ = 0.020

#### Refinement

*R*[*F* ^2^ > 2σ(*F* ^2^)] = 0.036
*wR*(*F* ^2^) = 0.102
*S* = 1.04
6905 reflections
327 parameters
H-atom parameters constrained
Δρ_max_ = 0.47 e Å^−3^
Δρ_min_ = −0.38 e Å^−3^

### 

Data collection: *APEX2* (Bruker, 2007 ▶); cell refinement: *SAINT* (Bruker, 2007 ▶); data reduction: *SAINT*; program(s) used to solve structure: *SHELXS97* (Sheldrick, 2008 ▶); program(s) used to refine structure: *SHELXL97* (Sheldrick, 2008 ▶); molecular graphics: *ORTEP-3* (Farrugia, 1997 ▶); software used to prepare material for publication: *SHELXL97*.

## Supplementary Material

Crystal structure: contains datablock(s) I, global. DOI: 10.1107/S1600536811045326/su2337sup1.cif

Structure factors: contains datablock(s) I. DOI: 10.1107/S1600536811045326/su2337Isup2.hkl

Supplementary material file. DOI: 10.1107/S1600536811045326/su2337Isup3.cml

Additional supplementary materials:  crystallographic information; 3D view; checkCIF report

## Figures and Tables

**Table 1:** Hydrogen-bond geometry (Å, °)

*D*—H⋯*A*	*D*—H	H⋯*A*	*D*⋯*A*	*D*—H⋯*A*
C7—H7*B*⋯O3^i^	0.97	2.59	3.511 (2)	158
C17—H17*B*⋯O1^ii^	0.97	2.58	3.516 (3)	164
C24—H24⋯Cl1^ii^	0.93	2.83	3.738 (2)	166

Symmetry codes: (i) 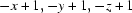; (ii) 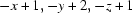.

**Fig. 1.**
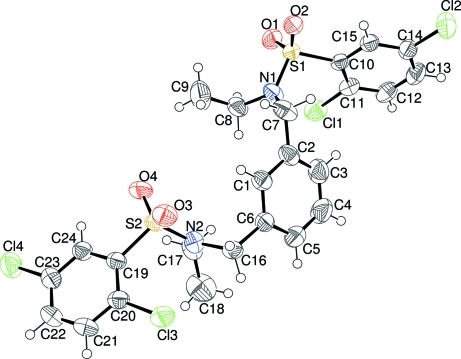
The molecular structure of the title molecule, showing 50% displacement ellipsoids and the numbering scheme.

## supplementary crystallographic information

### Comment

As part of our ongoing studies of symmetrical aryl sulfonamides (Ejaz *et al.*, 2011*a*,*b*), the synthesis and structure of the title compound are described herein.

In the title compound (Fig. 1), the dihedral angles between the central (C1-C6) benzene ring and the pendant (C10-C15) and (C19-C24) rings are 58.09 (1) and 62.59 (10)°, respectively. The equivalent angle between the pendant rings is 81.64 (9)°. Both sulfonamide groups lie to the same side of the central ring, but the C10—S1—N1—C7 and C19—S2—N2—C16 torsion angles [73.09 (16) and -117.35 (14)°, respectively] and the S1—N1—C7—C2 and S2—N2—C16—C6 torsion angles [-143.80 (14) and -111.45 (16)°, respectively] differ significantly for the two pendant chains. The conformations of the ethyl side chains are also different: the S1—N1—C8—C9 and S2—N2—C17—C18 torsion angles are -89.6 (2) and -126.57 (19)°, respectively. The nitrogen atoms are close to planar (bond angle sums = 356.4 and 359.5° for N1 and N2, respectively), which seems to indicate a valence state close to *sp*^2^ hybridization, as also seen in a related structure (Ejaz *et al.*, 2011*a*).

In the crystal, weak C—H···O and C—H···Cl interactions link the molecules (Table 1).

### Experimental

A mixture of *N*,*N*'-(benzene-1,3-diyldimethanediyl)bis(2,5-dichlorobenzenesulfonamide) (0.3 g; 0.5 mmol), sodium hydride (0.25 g; 0.9 mmol) and *N*,*N*-dimethylformamide (10.0 ml) was stirred in a 100-ml round bottom flask at room temperature for half an hour followed by the addition of ethyl iodide (0.15 g; 0.9 mmol). The reaction mixture was further stirred for five hours, and its completion was monitored by TLC. After completion, the contents were poured over crushed ice. The precipitated product was isolated, washed and crystallized from methanol to yield colourless block-like crystals of the title compound.

### Refinement

The hydrogen atoms were placed in calculated positions (C—H = 0.93–0.97 Å) and refined as riding atoms with *U*_iso_(H) = 1.2*U*_eq_(C) or 1.5*U*_eq_(methyl C). The methyl groups were allowed to rotate, but not to tip, to best fit the electron density.

### Crystal data

**Table:**

C_24_H_24_Cl_4_N_2_O_4_S_2_	*Z* = 2
*M**_r_* = 610.37	*F*(000) = 628
Triclinic, *P*1	*D*_x_ = 1.464 Mg m^−^^3^
Hall symbol: -P 1	Mo *K*α radiation, λ = 0.71073 Å
*a* = 8.0396 (2) Å	Cell parameters from 6905 reflections
*b* = 11.1512 (3) Å	θ = 2.2–28.1°
*c* = 15.5723 (3) Å	µ = 0.61 mm^−^^1^
α = 87.454 (1)°	*T* = 296 K
β = 83.378 (1)°	Chnnk, colourless
γ = 87.995 (1)°	0.50 × 0.35 × 0.30 mm
*V* = 1384.77 (6) Å^3^	

### Data collection

**Table:**

Bruker APEXII CCD diffractometer	5380 reflections with *I* > 2σ(*I*)
Radiation source: fine-focus sealed tube	*R*_int_ = 0.020
graphite	θ_max_ = 28.4°, θ_min_ = 3.0°
ω scans	*h* = −8→10
25271 measured reflections	*k* = −14→14
6905 independent reflections	*l* = −20→20

### Refinement

**Table:**

Refinement on *F*^2^	Primary atom site location: structure-invariant direct methods
Least-squares matrix: full	Secondary atom site location: difference Fourier map
*R*[*F*^2^ > 2σ(*F*^2^)] = 0.036	Hydrogen site location: inferred from neighbouring sites
*wR*(*F*^2^) = 0.102	H-atom parameters constrained
*S* = 1.04	*w* = 1/[σ^2^(*F*_o_^2^) + (0.0462*P*)^2^ + 0.4193*P*] where *P* = (*F*_o_^2^ + 2*F*_c_^2^)/3
6905 reflections	(Δ/σ)_max_ = 0.001
327 parameters	Δρ_max_ = 0.47 e Å^−^^3^
0 restraints	Δρ_min_ = −0.38 e Å^−^^3^

### Special details

**Table:**

Geometry. All e.s.d.'s (except the e.s.d. in the dihedral angle between two l.s. planes) are estimated using the full covariance matrix. The cell e.s.d.'s are taken into account individually in the estimation of e.s.d.'s in distances, angles and torsion angles; correlations between e.s.d.'s in cell parameters are only used when they are defined by crystal symmetry. An approximate (isotropic) treatment of cell e.s.d.'s is used for estimating e.s.d.'s involving l.s. planes.
Refinement. Refinement of *F*^2^ against ALL reflections. The weighted *R*-factor *wR* and goodness of fit *S* are based on *F*^2^, conventional *R*-factors *R* are based on *F*, with *F* set to zero for negative *F*^2^. The threshold expression of *F*^2^ > σ(*F*^2^) is used only for calculating *R*-factors(gt) *etc*. and is not relevant to the choice of reflections for refinement. *R*-factors based on *F*^2^ are statistically about twice as large as those based on *F*, and *R*- factors based on ALL data will be even larger.

### Fractional atomic coordinates and isotropic or equivalent isotropic displacement parameters (Å^2^)

**Table:**

	*x*	*y*	*z*	*U*_iso_*/*U*_eq_	
C1	0.6756 (2)	0.63163 (16)	0.43821 (11)	0.0454 (4)	
H1	0.5623	0.6242	0.4333	0.055*	
C2	0.7280 (3)	0.63672 (17)	0.51940 (12)	0.0502 (4)	
C3	0.8970 (3)	0.6480 (2)	0.52549 (15)	0.0695 (6)	
H3	0.9340	0.6525	0.5797	0.083*	
C4	1.0103 (3)	0.6527 (3)	0.45304 (17)	0.0776 (7)	
H4	1.1236	0.6594	0.4583	0.093*	
C5	0.9572 (3)	0.6475 (2)	0.37192 (14)	0.0622 (5)	
H5	1.0347	0.6509	0.3228	0.075*	
C6	0.7890 (2)	0.63737 (16)	0.36384 (11)	0.0452 (4)	
C7	0.6016 (3)	0.63174 (18)	0.59876 (12)	0.0579 (5)	
H7A	0.4935	0.6113	0.5825	0.069*	
H7B	0.6359	0.5696	0.6392	0.069*	
C8	0.5407 (3)	0.85534 (19)	0.58997 (13)	0.0571 (5)	
H8A	0.6171	0.8607	0.5371	0.069*	
H8B	0.5553	0.9256	0.6225	0.069*	
C9	0.3646 (4)	0.8577 (3)	0.5668 (2)	0.0967 (9)	
H9A	0.3463	0.9274	0.5304	0.145*	
H9B	0.2875	0.8603	0.6187	0.145*	
H9C	0.3473	0.7867	0.5367	0.145*	
C10	0.75316 (19)	0.69614 (16)	0.77750 (10)	0.0395 (3)	
C11	0.8994 (2)	0.75963 (17)	0.75576 (12)	0.0462 (4)	
C12	1.0466 (2)	0.71855 (19)	0.78590 (14)	0.0567 (5)	
H12	1.1438	0.7614	0.7717	0.068*	
C13	1.0506 (2)	0.6145 (2)	0.83684 (14)	0.0578 (5)	
H13	1.1499	0.5873	0.8574	0.069*	
C14	0.9069 (2)	0.55140 (17)	0.85701 (12)	0.0476 (4)	
C15	0.7572 (2)	0.59124 (16)	0.82812 (11)	0.0417 (4)	
H15	0.6605	0.5479	0.8426	0.050*	
C16	0.7269 (2)	0.63890 (16)	0.27608 (11)	0.0462 (4)	
H16A	0.8216	0.6338	0.2317	0.055*	
H16B	0.6586	0.5697	0.2724	0.055*	
C17	0.7074 (3)	0.86554 (18)	0.26202 (15)	0.0604 (5)	
H17A	0.7458	0.8739	0.3182	0.073*	
H17B	0.6251	0.9295	0.2538	0.073*	
C18	0.8521 (4)	0.8787 (3)	0.1935 (2)	0.0934 (9)	
H18A	0.8894	0.9598	0.1910	0.140*	
H18B	0.8181	0.8602	0.1386	0.140*	
H18C	0.9419	0.8246	0.2069	0.140*	
C19	0.4012 (2)	0.80340 (16)	0.14908 (10)	0.0414 (4)	
C20	0.4657 (2)	0.74445 (17)	0.07480 (11)	0.0470 (4)	
C21	0.4306 (3)	0.7893 (2)	−0.00540 (12)	0.0597 (5)	
H21	0.4722	0.7489	−0.0547	0.072*	
C22	0.3354 (3)	0.8928 (2)	−0.01335 (13)	0.0620 (5)	
H22	0.3119	0.9223	−0.0676	0.074*	
C23	0.2751 (2)	0.95234 (18)	0.05987 (12)	0.0514 (4)	
C24	0.3068 (2)	0.90861 (17)	0.14088 (11)	0.0458 (4)	
H24	0.2649	0.9497	0.1898	0.055*	
S1	0.55636 (5)	0.74464 (4)	0.74476 (3)	0.04106 (11)	
S2	0.42957 (6)	0.74915 (4)	0.25594 (3)	0.04402 (11)	
N1	0.5864 (2)	0.74805 (14)	0.64078 (9)	0.0489 (4)	
N2	0.62753 (19)	0.74925 (13)	0.26098 (10)	0.0475 (3)	
O1	0.51855 (17)	0.86403 (12)	0.77167 (9)	0.0560 (3)	
O2	0.44311 (14)	0.65269 (12)	0.77704 (8)	0.0515 (3)	
O3	0.37690 (17)	0.62781 (12)	0.26631 (9)	0.0563 (3)	
O4	0.34712 (19)	0.83655 (14)	0.31150 (8)	0.0622 (4)	
Cl1	0.90284 (7)	0.88984 (5)	0.69119 (4)	0.06287 (14)	
Cl2	0.91046 (7)	0.41825 (5)	0.91822 (4)	0.06768 (16)	
Cl3	0.59191 (7)	0.61629 (5)	0.07875 (3)	0.06285 (15)	
Cl4	0.15880 (8)	1.08554 (5)	0.05174 (4)	0.07195 (17)	

### Atomic displacement parameters (Å^2^)

**Table:**

	*U*^11^	*U*^22^	*U*^33^	*U*^12^	*U*^13^	*U*^23^
C1	0.0522 (10)	0.0428 (10)	0.0418 (9)	−0.0030 (7)	−0.0065 (7)	−0.0035 (7)
C2	0.0699 (12)	0.0418 (10)	0.0399 (9)	0.0025 (8)	−0.0106 (8)	−0.0043 (7)
C3	0.0735 (14)	0.0824 (16)	0.0571 (13)	0.0149 (12)	−0.0270 (11)	−0.0146 (11)
C4	0.0519 (12)	0.107 (2)	0.0773 (16)	0.0142 (12)	−0.0204 (11)	−0.0194 (14)
C5	0.0492 (11)	0.0776 (15)	0.0591 (12)	0.0081 (10)	−0.0047 (9)	−0.0072 (11)
C6	0.0494 (9)	0.0430 (10)	0.0429 (9)	0.0052 (7)	−0.0054 (7)	−0.0032 (7)
C7	0.0879 (15)	0.0477 (11)	0.0384 (9)	−0.0080 (10)	−0.0066 (9)	−0.0019 (8)
C8	0.0692 (13)	0.0528 (11)	0.0463 (10)	0.0054 (9)	−0.0006 (9)	0.0103 (9)
C9	0.0856 (19)	0.106 (2)	0.099 (2)	0.0127 (16)	−0.0304 (16)	0.0276 (18)
C10	0.0340 (7)	0.0486 (10)	0.0360 (8)	−0.0002 (7)	−0.0017 (6)	−0.0088 (7)
C11	0.0416 (9)	0.0473 (10)	0.0486 (10)	−0.0037 (7)	0.0021 (7)	−0.0083 (8)
C12	0.0345 (8)	0.0634 (13)	0.0716 (13)	−0.0056 (8)	−0.0007 (8)	−0.0081 (10)
C13	0.0368 (9)	0.0688 (13)	0.0688 (13)	0.0047 (8)	−0.0101 (8)	−0.0098 (10)
C14	0.0446 (9)	0.0508 (11)	0.0478 (10)	0.0054 (8)	−0.0077 (7)	−0.0067 (8)
C15	0.0363 (8)	0.0506 (10)	0.0383 (8)	−0.0013 (7)	−0.0034 (6)	−0.0068 (7)
C16	0.0526 (10)	0.0469 (10)	0.0382 (9)	0.0034 (8)	−0.0012 (7)	−0.0060 (7)
C17	0.0711 (13)	0.0451 (11)	0.0688 (13)	−0.0084 (9)	−0.0234 (11)	0.0018 (9)
C18	0.098 (2)	0.0845 (19)	0.097 (2)	−0.0371 (16)	−0.0047 (16)	0.0179 (16)
C19	0.0412 (8)	0.0490 (10)	0.0340 (8)	−0.0053 (7)	−0.0023 (6)	−0.0035 (7)
C20	0.0479 (9)	0.0528 (11)	0.0394 (9)	−0.0017 (8)	0.0001 (7)	−0.0069 (8)
C21	0.0703 (13)	0.0731 (14)	0.0347 (9)	0.0004 (11)	−0.0004 (8)	−0.0085 (9)
C22	0.0715 (13)	0.0772 (15)	0.0375 (10)	0.0008 (11)	−0.0103 (9)	0.0033 (9)
C23	0.0531 (10)	0.0530 (11)	0.0486 (10)	−0.0030 (8)	−0.0100 (8)	0.0023 (8)
C24	0.0452 (9)	0.0519 (11)	0.0406 (9)	−0.0028 (8)	−0.0044 (7)	−0.0054 (8)
S1	0.0364 (2)	0.0499 (3)	0.0361 (2)	0.00241 (17)	−0.00185 (15)	−0.00196 (17)
S2	0.0484 (2)	0.0491 (3)	0.0329 (2)	−0.00132 (18)	0.00197 (16)	−0.00125 (17)
N1	0.0683 (10)	0.0431 (8)	0.0353 (7)	0.0015 (7)	−0.0079 (7)	0.0006 (6)
N2	0.0529 (8)	0.0411 (8)	0.0501 (8)	−0.0039 (6)	−0.0123 (7)	−0.0002 (6)
O1	0.0568 (8)	0.0580 (8)	0.0521 (8)	0.0143 (6)	−0.0022 (6)	−0.0118 (6)
O2	0.0339 (6)	0.0686 (9)	0.0508 (7)	−0.0053 (6)	−0.0034 (5)	0.0098 (6)
O3	0.0571 (8)	0.0549 (8)	0.0549 (8)	−0.0138 (6)	0.0022 (6)	0.0074 (6)
O4	0.0727 (9)	0.0730 (10)	0.0378 (7)	0.0149 (7)	0.0046 (6)	−0.0090 (6)
Cl1	0.0584 (3)	0.0556 (3)	0.0720 (3)	−0.0115 (2)	0.0042 (2)	0.0037 (2)
Cl2	0.0640 (3)	0.0647 (3)	0.0758 (4)	0.0053 (2)	−0.0212 (3)	0.0105 (3)
Cl3	0.0713 (3)	0.0645 (3)	0.0507 (3)	0.0141 (3)	0.0007 (2)	−0.0128 (2)
Cl4	0.0829 (4)	0.0666 (4)	0.0675 (3)	0.0136 (3)	−0.0205 (3)	0.0039 (3)

### Geometric parameters (Å, °)

**Table:**

C1—C2	1.382 (2)	C14—C15	1.385 (2)
C1—C6	1.389 (3)	C14—Cl2	1.730 (2)
C1—H1	0.9300	C15—H15	0.9300
C2—C3	1.383 (3)	C16—N2	1.468 (2)
C2—C7	1.507 (3)	C16—H16A	0.9700
C3—C4	1.366 (3)	C16—H16B	0.9700
C3—H3	0.9300	C17—N2	1.469 (2)
C4—C5	1.383 (3)	C17—C18	1.491 (4)
C4—H4	0.9300	C17—H17A	0.9700
C5—C6	1.381 (3)	C17—H17B	0.9700
C5—H5	0.9300	C18—H18A	0.9600
C6—C16	1.507 (2)	C18—H18B	0.9600
C7—N1	1.474 (2)	C18—H18C	0.9600
C7—H7A	0.9700	C19—C24	1.384 (2)
C7—H7B	0.9700	C19—C20	1.394 (2)
C8—N1	1.465 (2)	C19—S2	1.7820 (17)
C8—C9	1.499 (3)	C20—C21	1.380 (3)
C8—H8A	0.9700	C20—Cl3	1.726 (2)
C8—H8B	0.9700	C21—C22	1.371 (3)
C9—H9A	0.9600	C21—H21	0.9300
C9—H9B	0.9600	C22—C23	1.376 (3)
C9—H9C	0.9600	C22—H22	0.9300
C10—C15	1.383 (2)	C23—C24	1.380 (3)
C10—C11	1.394 (2)	C23—Cl4	1.735 (2)
C10—S1	1.7769 (16)	C24—H24	0.9300
C11—C12	1.379 (3)	S1—O1	1.4256 (14)
C11—Cl1	1.7282 (19)	S1—O2	1.4307 (13)
C12—C13	1.378 (3)	S1—N1	1.6082 (15)
C12—H12	0.9300	S2—O4	1.4267 (14)
C13—C14	1.372 (3)	S2—O3	1.4286 (14)
C13—H13	0.9300	S2—N2	1.6025 (16)
			
C2—C1—C6	121.26 (18)	N2—C16—C6	110.74 (14)
C2—C1—H1	119.4	N2—C16—H16A	109.5
C6—C1—H1	119.4	C6—C16—H16A	109.5
C1—C2—C3	118.56 (19)	N2—C16—H16B	109.5
C1—C2—C7	119.97 (19)	C6—C16—H16B	109.5
C3—C2—C7	121.47 (18)	H16A—C16—H16B	108.1
C4—C3—C2	120.9 (2)	N2—C17—C18	112.5 (2)
C4—C3—H3	119.6	N2—C17—H17A	109.1
C2—C3—H3	119.6	C18—C17—H17A	109.1
C3—C4—C5	120.3 (2)	N2—C17—H17B	109.1
C3—C4—H4	119.8	C18—C17—H17B	109.1
C5—C4—H4	119.8	H17A—C17—H17B	107.8
C6—C5—C4	120.1 (2)	C17—C18—H18A	109.5
C6—C5—H5	120.0	C17—C18—H18B	109.5
C4—C5—H5	120.0	H18A—C18—H18B	109.5
C5—C6—C1	118.91 (17)	C17—C18—H18C	109.5
C5—C6—C16	121.04 (17)	H18A—C18—H18C	109.5
C1—C6—C16	119.97 (16)	H18B—C18—H18C	109.5
N1—C7—C2	110.85 (16)	C24—C19—C20	119.20 (16)
N1—C7—H7A	109.5	C24—C19—S2	117.24 (13)
C2—C7—H7A	109.5	C20—C19—S2	123.55 (14)
N1—C7—H7B	109.5	C21—C20—C19	119.87 (18)
C2—C7—H7B	109.5	C21—C20—Cl3	117.85 (15)
H7A—C7—H7B	108.1	C19—C20—Cl3	122.28 (14)
N1—C8—C9	114.22 (19)	C22—C21—C20	120.85 (19)
N1—C8—H8A	108.7	C22—C21—H21	119.6
C9—C8—H8A	108.7	C20—C21—H21	119.6
N1—C8—H8B	108.7	C21—C22—C23	119.20 (18)
C9—C8—H8B	108.7	C21—C22—H22	120.4
H8A—C8—H8B	107.6	C23—C22—H22	120.4
C8—C9—H9A	109.5	C22—C23—C24	121.06 (19)
C8—C9—H9B	109.5	C22—C23—Cl4	120.31 (15)
H9A—C9—H9B	109.5	C24—C23—Cl4	118.63 (16)
C8—C9—H9C	109.5	C23—C24—C19	119.79 (17)
H9A—C9—H9C	109.5	C23—C24—H24	120.1
H9B—C9—H9C	109.5	C19—C24—H24	120.1
C15—C10—C11	119.74 (15)	O1—S1—O2	118.08 (8)
C15—C10—S1	117.14 (12)	O1—S1—N1	108.23 (8)
C11—C10—S1	123.11 (14)	O2—S1—N1	110.99 (8)
C12—C11—C10	119.87 (18)	O1—S1—C10	109.11 (8)
C12—C11—Cl1	118.30 (15)	O2—S1—C10	105.20 (8)
C10—C11—Cl1	121.83 (14)	N1—S1—C10	104.35 (8)
C13—C12—C11	120.47 (18)	O4—S2—O3	118.77 (9)
C13—C12—H12	119.8	O4—S2—N2	109.82 (9)
C11—C12—H12	119.8	O3—S2—N2	108.25 (8)
C14—C13—C12	119.45 (18)	O4—S2—C19	105.17 (9)
C14—C13—H13	120.3	O3—S2—C19	108.50 (8)
C12—C13—H13	120.3	N2—S2—C19	105.53 (8)
C13—C14—C15	121.21 (18)	C8—N1—C7	118.50 (15)
C13—C14—Cl2	120.22 (15)	C8—N1—S1	120.83 (13)
C15—C14—Cl2	118.56 (15)	C7—N1—S1	117.12 (12)
C10—C15—C14	119.25 (16)	C16—N2—C17	118.81 (16)
C10—C15—H15	120.4	C16—N2—S2	122.54 (12)
C14—C15—H15	120.4	C17—N2—S2	118.14 (13)
			
C6—C1—C2—C3	0.1 (3)	C22—C23—C24—C19	0.2 (3)
C6—C1—C2—C7	179.28 (17)	Cl4—C23—C24—C19	−179.11 (14)
C1—C2—C3—C4	−0.7 (3)	C20—C19—C24—C23	1.3 (3)
C7—C2—C3—C4	−179.9 (2)	S2—C19—C24—C23	−177.43 (14)
C2—C3—C4—C5	0.7 (4)	C15—C10—S1—O1	124.12 (14)
C3—C4—C5—C6	−0.1 (4)	C11—C10—S1—O1	−54.76 (16)
C4—C5—C6—C1	−0.5 (3)	C15—C10—S1—O2	−3.50 (15)
C4—C5—C6—C16	176.4 (2)	C11—C10—S1—O2	177.63 (14)
C2—C1—C6—C5	0.5 (3)	C15—C10—S1—N1	−120.40 (14)
C2—C1—C6—C16	−176.41 (17)	C11—C10—S1—N1	60.73 (16)
C1—C2—C7—N1	−111.1 (2)	C24—C19—S2—O4	−2.36 (16)
C3—C2—C7—N1	68.0 (2)	C20—C19—S2—O4	178.98 (15)
C15—C10—C11—C12	−1.0 (3)	C24—C19—S2—O3	125.71 (14)
S1—C10—C11—C12	177.85 (15)	C20—C19—S2—O3	−52.96 (17)
C15—C10—C11—Cl1	178.88 (13)	C24—C19—S2—N2	−118.46 (14)
S1—C10—C11—Cl1	−2.3 (2)	C20—C19—S2—N2	62.87 (17)
C10—C11—C12—C13	0.5 (3)	C9—C8—N1—C7	68.6 (3)
Cl1—C11—C12—C13	−179.33 (16)	C9—C8—N1—S1	−89.6 (2)
C11—C12—C13—C14	0.4 (3)	C2—C7—N1—C8	57.3 (2)
C12—C13—C14—C15	−0.9 (3)	C2—C7—N1—S1	−143.80 (14)
C12—C13—C14—Cl2	178.11 (16)	O1—S1—N1—C8	−12.41 (18)
C11—C10—C15—C14	0.5 (3)	O2—S1—N1—C8	118.67 (16)
S1—C10—C15—C14	−178.43 (13)	C10—S1—N1—C8	−128.51 (16)
C13—C14—C15—C10	0.5 (3)	O1—S1—N1—C7	−170.82 (15)
Cl2—C14—C15—C10	−178.57 (13)	O2—S1—N1—C7	−39.74 (17)
C5—C6—C16—N2	−111.2 (2)	C10—S1—N1—C7	73.09 (16)
C1—C6—C16—N2	65.6 (2)	C6—C16—N2—C17	60.2 (2)
C24—C19—C20—C21	−2.0 (3)	C6—C16—N2—S2	−111.45 (16)
S2—C19—C20—C21	176.64 (16)	C18—C17—N2—C16	61.4 (2)
C24—C19—C20—Cl3	177.23 (14)	C18—C17—N2—S2	−126.57 (19)
S2—C19—C20—Cl3	−4.1 (2)	O4—S2—N2—C16	129.76 (14)
C19—C20—C21—C22	1.2 (3)	O3—S2—N2—C16	−1.35 (16)
Cl3—C20—C21—C22	−178.04 (17)	C19—S2—N2—C16	−117.35 (14)
C20—C21—C22—C23	0.3 (3)	O4—S2—N2—C17	−41.94 (17)
C21—C22—C23—C24	−1.0 (3)	O3—S2—N2—C17	−173.06 (14)
C21—C22—C23—Cl4	178.31 (17)	C19—S2—N2—C17	70.94 (16)

### Hydrogen-bond geometry (Å, °)

**Table:**

*D*—H···*A*	*D*—H	H···*A*	*D*···*A*	*D*—H···*A*
C7—H7B···O3^i^	0.97	2.59	3.511 (2)	158
C17—H17B···O1^ii^	0.97	2.58	3.516 (3)	164
C24—H24···Cl1^ii^	0.93	2.83	3.738 (2)	166

Symmetry codes: (i) −*x*+1, −*y*+1, −*z*+1; (ii) −*x*+1, −*y*+2, −*z*+1.
